# Novel Molecular Mechanisms of Pulmonary Hypertension: A Search for Biomarkers and Novel Drug Targets—From Bench to Bed Site

**DOI:** 10.1155/2020/7265487

**Published:** 2020-05-22

**Authors:** Damian Gajecki, Jakub Gawrys, Ewa Szahidewicz-Krupska, Adrian Doroszko

**Affiliations:** Department of Internal Medicine, Hypertension and Clinical Oncology, Faculty of Medicine, Wroclaw Medical University, Wroclaw, Poland

## Abstract

Pulmonary hypertension (PH) is defined as increased mean pulmonary artery pressure (mPAP) above 25 mmHg, measured at rest by right heart catheterization. The exact global prevalence of PH is difficult to estimate, mainly due to the complex aetiology, and its spread may be underestimated. To date, numerous studies on the aetiology and pathophysiology of PH at molecular level were conducted. Simultaneously, some clinical studies have shown potential usefulness of well-known and widely recognized cardiovascular biomarkers, but their potential clinical usefulness in diagnosis and management of PH is poor due to their low specificity accompanied with numerous other cardiovascular comorbidities of PH subjects. On the other hand, a large body of basic research-based studies provides us with novel molecular pathomechanisms, biomarkers, and drug targets, according to the evidence-based medicine principles. Unfortunately, the simple implementation of these results to clinical practice is impossible due to a large heterogeneity of the PH pathophysiology, where the clinical symptoms constitute only a common denominator and a final result of numerous crosstalking metabolic pathways. Therefore, future studies, based mostly on translational medicine, are needed in order to both organize better the pathophysiological classification of various forms of PH and define precisely the optimal diagnostic markers and therapeutic targets in particular forms of PH. This review paper summarizes the current state of the art regarding the molecular background of PH with respect to its current classification. Novel therapeutic strategies and potential biomarkers are discussed with respect to their limitations in use in common clinical practice.

## 1. Introduction

Pulmonary hypertension (PH) is defined as increased mean pulmonary arterial pressure (mPAP) above 25 mmHg, measured at rest by right heart catheterization [1]. The exact global prevalence of the disease is difficult to estimate mainly due to the complex aetiology, and its spread may be significantly underestimated.

Based on the hemodynamic parameters assessed during right heart catheterization (especially DPG (diastolic pressure gradient) and PVR (pulmonary vascular resistance)), PH was divided into pre- and postcapillary PH. Postcapillary PH occurs as isolated or combined pre- and postcapillary PH. Additionally, taking under consideration clinical assessment, pathophysiology, pathological similarities, and treatment approaches, the PH patients were categorized into 5 groups with concurrent subgroups (Table 1) [2, 3].

It is currently assumed that prevalence of PH is around 0.3% in general population, although some studies estimate it to 6.6% [4, 5]. Pulmonary hypertension is more common in women than in men (1.8 : 1.0), and the incidence increases with age.

Pulmonary hypertension is characterized by a complex aetiology. The pathophysiological mechanisms leading to increased pressure in the pulmonary vessels are primarily connected with vascular remodelling. They can be caused by primary dysfunctions of endothelial cells (ECs) or smooth muscles accompanied by proliferative disorders, oxidative damage, abnormal angiogenesis, or capillary leak. Vascular remodelling can also occur secondarily to vascular overload associated with a retrograde passive transmission of elevated venous pressure (i.e., in left-sided heart diseases), mechanical narrowing of pulmonary arteries by embolic material, impaired immune processes, and hypoxia-associated vasoconstriction. An important role is also played by the Euler-Liljestrand reflex, in which the presence of alveolar hypoxia causes vasoconstriction and blood redistribution to better oxygenize parts of the pulmonary vascular. Such condition, augmented by the imbalance between vasoconstricting and vasodilating factors, leads to a cascade of abnormalities that exacerbate each other (a vicious circle). Numerous signalling pathways and signalling molecules participate in these phenomena, which finally leads to functional and structural changes in the pulmonary vessels [2].

This review summarizes the state of the art regarding the knowledge on the pathomechanisms of PH. We also discuss some data from multiomic studies that could provide new biomarkers reflecting the burden of the pulmonary vascular remodelling and defining some novel drug targets.

## 2. Genetic Mutations as a Cause of Familial Pulmonary Arterial Hypertension (PAH)

### 2.1. TGF-*β* Family Protein Signalling Pathway Dysfunction in PAH

Familial pulmonary hypertension occurs in approximately 3.2% of patients with pulmonary hypertension [6]. The signalling pathway has an important role in the regulation of physiological changes in the pulmonary vascular endothelium, transmitted through the receptor proteins of the TGF-*β* family, which modulate the transcription and translation of numerous genes responsible, among others, for regulating the inflammatory response, cell proliferation, and differentiation. Disruption of this pathway may cause abnormal vascular remodelling, exacerbate atherosclerosis, and affect myocardial fibrosis.

Factors from the TGF-*β* signalling family regulate cell metabolism by transducing the signal through the transmembrane complex. This structure consists of two type II receptors and two type I receptors with serine-threonine kinase activity. In mammals, seven types of receptor I (ALK1-7), five types of receptor II (including but not limited to BMPR2), and two types of receptor III (endoglin and beta-glycan) have been characterized. As a result of receptor stimulation by an appropriate ligand, phosphorylation of intracellular substrates occurs and signal is transmitted to the cell nucleus through receptor-regulated SMAD proteins (R-SMAD, which include SMAD1-3, SMAD5, and SMAD8). They form a complex with the SMAD4 protein and thus transfer the signal inside the cell's nucleus. This pathway is known as the canonical BMP signalling.

In the pathogenesis of pulmonary hypertension, the main abnormalities are connected with mutations altering the properties of ALK1 and ALK5, BMPR2 and endoglin receptors, and SMAD8 signalling proteins (they are responsible for inhibiting signalling by R-SMAD) [7–10].

A series of studies report that the loss of signal mediated by SMAD1/5/8 plays an important role in the pulmonary vascular remodelling and the pathogenesis of PAH. Functional studies on BMPR2 mutations have shown that BMPR2 mutations disrupted or downregulated SMAD1/5/8 signalling.

Shintani et al. identified a nonsense mutation in the SMAD8 gene (also known as SMAD9) which introduces a premature stop codon into exon 2 and results in a truncated protein, not phosphorylated by the constitutively active ALK3 and ALK1 proteins. As a result, the interaction with SMAD4 is impossible, the signal is not transmitted into the nucleus, and the transcription of the target genes is not downregulated [11].

Yang et al. [12] revealed that BMP4 caused either inhibition or stimulation of PASMC cell proliferation, depending on cell origin. Thus, normal PASMCs isolated from the proximal pulmonary artery were inhibited, whereas normal PASMCs isolated from peripheral arteries were stimulated to proliferate in the presence of BMP4. It was demonstrated that BMP4 causes phosphorylation of SMAD1/6/7 and activates the canonical BMP signalling and, at the same time, the SMAD-independent p38MAPK and ERK1/2 pathways.

In the further part of the said study, Yang et al. [12] pointed out a predominant role of SMAD1-dependent growth suppression and indicated the proproliferative p38MAPK- and ERK1/2-dependent pathways in PASMCs in response to BMP4 activation. Using the p38 and ERK1/2 inhibitors, the authors prove that these proteins have a significant role in distal vessel PASMC proliferation. In other studies, it was revealed that the presence of a mutant BMPR2 receptor led to constitutive activation of p38MAPK even in the absence of ligand stimulation [13]. Moreover, it was indicated that in cultured cells from BMPR2-mutated patients, dysfunction of SMAD1 leads to an antiproliferation defect which may be amplified by p38MAPK inhibition.

These observations may indicate potential new targets in the therapy of FPAH patients, using agents that could inhibit the abnormal proliferation in PASMCs. Disruption of the p38MAPK signalling pathway may be a new goal of antiproliferative strategies to prevent or reverse the remodelling associated with the BMPR2 mutation.

Gore et al. showed that the expression of TGF-*β* family proteins is significantly higher in lung tissue and plasma of patients suffering from idiopathic pulmonary hypertension. In addition, higher concentration of TGF-*β* mRNA was determined in pulmonary vascular smooth muscle. They also demonstrated that endothelial cells treated with serum, where previously other ECs stimulated by TGF-*β* were grown, resulted in increased proliferation rate of the former. It confirms that previously postulated vascular muscle proliferation is dependent on factors secreted by endothelial cells, which is further intensified by TGF-*β* stimulation. The increase in muscle proliferation by TGF-*β* was abolished when the antiendoglin antibodies were added [7].

Noteworthily, significant amounts of endoglin and ALK1 encoding mRNA and the proteins themselves were determined in ECs. Other studies have shown that endoglin (CD105) plays a key role in maintaining the balance of signal pathways transmitted by the ALK1 and ALK5 receptors. Thus, it regulates vascular epithelial proliferation and its presence is shown on the surface of dividing endothelial cells in vivo [14].

However, the role of the TGF-*β*/ALK pathway in the pathogenesis of pulmonary hypertension is not clearly defined. Similar to the research of Gore et al., the ALK1 overexpression in studies, in the animal model of monocrotaline-induced pulmonary hypertension, was shown by Ramos et al. [15] However, a study conducted on a similar model by Zakrzewicz et al. showed a decrease in expression of the TGF-*β* pathway proteins [16]. The concentration of other circulating ligands, such as activins and growth and differentiation factors (GDFs), which also interact through the TGF-*β* receptor family, is also increased in PAH, which probably stimulates growth cells, and thus contributes to the remodelling of pulmonary vessels and is an additional pathomechanism for the development of pulmonary hypertension [10]. However, the importance of these processes requires further study.

Mutations in the genes encoding the TGF-*β* family signalling pathways have also been shown to cause pulmonary hypertension associated with Osler-Weber-Rendu disease. Most of them were detected only in individual patients and relate to mutations in ENG genes (encoding endoglin) 61% and ACVRL1 (encoding receptor-like activin kinase type 1 (ALK1)) 37% [17]. Other mutations were also revealed in MADH4 genes (encoding SMAD4) and genes localized on the 5q and 7p chromosomes [18, 19].

### 2.2. BMPR2 Mutation in PAH

The best-known cause of hereditary pulmonary hypertension is a mutation in the bone morphogenetic protein receptor type 2 (BMPR2) gene, which belongs to the TGF-*β* superfamily. It is detected in 55% of patients with familial pulmonary arterial hypertension (PAH) and in at least 26% of patients with idiopathic pulmonary hypertension [20, 21]. Initially, it was found that this protein is responsible for regulating bone and cartilage growth and differentiation, hence its name. Further studies have shown that it also regulates the proliferation, apoptosis, differentiation, and migration of pulmonary artery cells. As a result, excessive proliferation in pulmonary muscle cells (PMCs) occurs, which results in overload and subsequent right ventricular failure with the simultaneous development of pulmonary hypertension [22]. BMPR2 gene mutation has been shown to present haploinsufficiency, so one remaining functional copy of the gene does not allow to produce competent protein and results in disruption of biological function and reduction of apoptotic response of pulmonary smooth muscle cells compared to controls [21, 23].

It is estimated that about 15-20% of patients with the BMPR2 gene mutation will develop pulmonary hypertension [24]. Girerd et al. pointed out that patients with the BMPR2 mutation are significantly younger at the moment of diagnosis compared to patients in which no such mutation was found (38.53 ± 12.38 vs. 45.78 ± 11.32 years, *P* < 0.001) [25, 26]. It was indicated that, as in the entire group of PH, also in BMPR2 mutant population, women dominate (female : male ratio 2.4 : 1) [26]. It is postulated that the difference may be due to effect of oestrogen on vascular cells. It results in abnormal cell division and vasoreactivity inhibition. In vitro, oestrogens increase the proliferation of epithelial smooth muscle cells, which is inhibited by antioestrogen drugs, e.g., tamoxifen.

It is worth noting that the most important enzyme regulating oestrogen metabolism is cytochrome P450 isoform 1B, which is highly expressed in oestrogen-dependent tissues, as well as in lung tissue, where it can be responsible for regulating oestrogen balance. West et al. revealed lower transcript concentration of CYP1B1 in women with pulmonary hypertension. This may lead to higher local oestrogen levels and an increased risk of PAH [27]. In addition, lower enzyme levels may also result in oestrogen transformation, by other enzymes, into more potent compounds with higher PH inducing feature [28].

BMPR2 mutation carriers have been shown to be characterized by higher mean pulmonary artery pressure (mPAP) and pulmonary vascular resistance (PVR), are less likely to receive a positive response in an acute hemodynamic test, and more often exacerbate a fatal disease or require lung transplantation. However, no significant differences in the survival were found [25]. In the other studies, Austin et al. did not reveal the same statistical significance in the marked hemodynamic parameters in 169 healthy patients with a mutation in the BMPR2 gene [29].

Austin et al. pointed out also that the BMPR2 missense mutation was linked with significantly worse outcomes and the disease has a more severe clinical course (age at the time of diagnosis, shorter time to lung transplant or death), compared to the group with nonsense BPMR2 mutation. Such correlation was not confirmed by Girerd et al. in other study [26, 29].

Noteworthily, some papers highlight that the BMPR2 mutant men present worse prognosis and higher mortality than female mutation carriers; however, further research studies are needed to confirm this observation [26, 30].

### 2.3. Caveolin-1 Mutation in PAH

Caveolin-1 is a scaffolding protein that is encoded by the *CAV1* gene and constitutes a part of the plasma membrane contributing to formation of caveola subtype of specialized microdomains known as lipid rafts. They are abundant of many receptors on the surface and are the point where many signalling cascades start. It has been shown that caveolin-1 interacts with, e.g., G proteins, TGF-*β* receptor 1, endothelial nitric oxide synthase (eNOS), and nitric oxide synthase 2A [31–33].

Zhao et al. [34, 35] used genetically changed mice with CAV1 deletion to verify the role of caveolin in the pathogenesis of PAH. They reported that mutated mice have presented increased activity of eNOS, which leads to vascular remodelling and PH phenotype. It was proven that according to induced oxidative stress, superoxide and nitric oxide (NO) react to form peroxynitrite, which in turn causes increased protein kinase G (PKG) tyrosine nitration and disrupts its appropriate function resulting in PAH development. Interestingly, high levels of NO *per se* in CAV1–/– mice do not cause PH since production of superoxide is critically important [36].

Interestingly, the same study also proved that CAV1–/– mice were protected from developing the changes in pulmonary arteries while being treated with superoxide scavenger, with NOS inhibitor, or by adenovirus-mediated restoration of PKG activity in CAV1–/– lungs. Furthermore, double-knockout mice, deficient in CAV1 and eNOS (Nos3), also did not present the PAH phenotype [35].

In another study, Courchamp et al. found that in CAV1–/– mice, a lack of caveolae causes severe pathomorphological defects in the alveolar septum. They proved that normal double-layered alveolar architecture was replaced by multilayered, unorganized tissue. In addition, uncontrolled endothelial cell proliferation and fibrosis have been observed [36].

### 2.4. KCNK3 Mutation in PAH

The KCNK3 gene encodes an outward K+ channel that is sensitive to changes in extracellular PH and is inhibited by extracellular acidification. Accordingly, it is also called Twik-related acid-sensitive K+ channel (TASK1). The primary role of KCNK3 channels is to control the resting membrane potential in many cell types, including human PMCs. They take part in relaxing the arteries through the action of smooth muscle cells [37].

It was reported that KCNK3 expression and function are incomplete in patients with idiopathic hereditary pulmonary arterial hypertension and in the PAH rat model induced by monocrotaline. To confirm that defective potassium channels lead to PAH phenotype, the KCNK3-selective KCNK3 blocker was used. After the long-term blockade, they demonstrated distal artery neomuscularization and increased proliferation of PAECs, PASMCs, and inflammation level. Therefore, the important role of KCNK3 in downregulating cell proliferation and modulation of pulmonary arterial tone is postulated [38].

Ma et al. carried out the whole-exome sequencing in a family with multiple members suffering from pulmonary arterial hypertension without identifiable mutations known to be associated with PAH and revealed five novel mutations in KCNK3 genes which resulted in a loss of function and reduction in potassium current which was confirmed electrophysiologically. They proved that channel function can be restored to a variable extent using the phospholipase A2 inhibitor (ONO-RS-082). Moreover, administration of prostacyclin analogue, treprostinil, or cAMP analogue, 8-bromo-cAMP, also causes KCNK3 activation. Similar conclusions were made in other studies where KCNK3 activation reduces the development of PH in the monocrotaline rat model. This approach modifying the receptor may constitute an interesting therapeutic strategy for human PAH. These studies highlight the significant progress made in using genetic testing in PAH patient care [39].

### 2.5. EIF2AK4 Mutation in Pulmonary Veno-occlusive Disease (PVOD) and/or Pulmonary Capillary Hemangiomatosis (PCH)

The eukaryotic translation initiation factor 2 alpha kinase 4 (EIF2AK4) is a serine-threonine kinase that phosphorylates the alpha subunit of eukaryotic translation initiation factor 2 alpha (eIF2*α*) and changes gene expression in response to cellular stress. To date, EIF2AK4 mutations were identified in all familial PVOD/PCH genetically tested individuals and in 20-25% sporadic cases [40, 41].

EIF2AK4 regulates angiogenesis by altering gene expression in response to stress factors, mainly amino acid deficiency. It also participates in the cellular response caused by viral infection, glucose deficiency, or UV exposure. In response to hypoxia, EIF2AK4 reduces cell proliferation, thereby protecting against hypoxia-induced remodelling [42].

Chaveroux et al. evaluated the role of EIF2AK4 in maintaining homeostasis in oxidative stress. The authors exposed EIF2AK4–/– mice to imbalanced diet, partially depleted for leucine, and evaluated levels of oxidized proteins in mouse livers. They indicated increased oxidative stress, one of the well-established factors in PH pathogenesis proving thus that EIF2AK4 activity is necessary to resist oxidative stress caused by leucine deficiency [43].

Eichstaedt et al. studied the role of EIF2AK4 mutation as a potential reason of incomplete penetrance in the family with BMPR2 mutations. They conducted Sanger sequencing in all living members of the family burdened with HPAH caused by the BMPR2 mutation. They were looking for other mutations that could affect incomplete penetrance and reported that all family members who were suffering from pulmonary hypertension (confirmed with right heart catheterization) had an additional mutation in the EIF2AK4 gene. The results may support the hypothesis of a second hit model where a single mutation is linked with low penetrance; however, two independent mutations lead to a synergistic effect resulting in full-blown disease [44]. The occurrence of several mutations in genes related to PAH may be more frequent than originally thought.

Moreover, animal studies point out that the EIF2AK4/eIF2*α*/ATF4 pathway leads to induction of the TRB3 gene transcription in response to a leucine-deficient diet. TRB3 is considered to be responsible for regulating the BMP signalling pathway through modulation of the Smurf1 protein level by changing its ubiquitination and degradation. The decrease in TRB3 concentration in the case of the EIF2AK4 mutation may therefore lead to imbalance of Smurf1 ubiquitination. It may eventually interfere with TGF-*β* family transmission and be another reason exacerbating pulmonary hypertension development in the case of BMPR2 haploinsufficiency [45].

What is more, in the Barrios-Rodiles et al. study, the authors analyzed the dynamic protein-protein interaction networks in the TGF-*β* pathway and found EIF2AK4 to interact with SMAD4, SMAD1, ALK1, and endoglin (ENG) [46].

However, further research is necessary to determine the importance of these interactions in the pathogenesis of pulmonary hypertension.

The availability of molecular genetic diagnosis has opened up a new field for patient care, including genetic counselling. However, in spite of multidirectional genetic tests, to date, no clear therapeutic approach has been defined for patients with PAH. A large group of patients, despite the optimal currently available therapy, finally undergoes lung transplantation.

## 3. Epigenetic Modifications in Pulmonary Hypertension

The analysis of PAH patients' families indicates that the occurrence of the disease and its severity is modulated by additional factors besides the genetic mutations themselves. The epigenetic modifications could explain the nature of the PAH clinical course.

### 3.1. DNA Methylation

To date, the best studied epigenetic modification is the DNA methylation. As it is well known, the level of the gene expression is dependent on the promoter methylation degree. The greater the degree of promoter methylation, the stronger the transcription repression of a given gene. The addition of the methyl groups is catalyzed by DNMT (DNA methyltransferase) enzymes (including DNMT1, DNMT3A, and DNMT3B). There are at least two mechanisms, by which methylation blocks gene expression. In the first, the addition of a methyl group changes the spatial structure of the DNA sequence and prevents the transcription factor from attaching. In the second mechanism, methylation leads to the attachment of specific proteins, which prevents transcription factors from accessing chromatin. Both processes lead to transcription blockage [47].

Napoli et al. indicated that the BMPR2 promoter is significantly more methylated in patients with HPAH compared to controls. This epigenetic modification leads to PAH phenotype even in heterozygous mutant patients. It is known along with the Gimelbrant et al. study that only one of the alleles—maternal or paternal—is expressed in the cell. That is why the hypermethylation of wild allele in heterozygous proband may cause null mutation and, as a result, change the penetration of PAH in affected families leading to the early onset of the disease. Using drugs that change the methylation level of the BMPR promoter may be a potential therapeutic strategy for HPAH [47–49].

The other already-found hypermethylated gene in a patient with PAH is ABCA1. It is responsible for coding the proteins that transport cholesterol and phospholipids across the cell membrane to form the high-density lipoprotein (HDL). In addition to this, it was revealed that evaluation of ABCA1 methylation may become a useful biomarker to separate patient at risk of PAH [47].

### 3.2. Modifications of MicroRNA (miRNA)

The other dysfunctional mechanisms in PAH are microRNA (miRNA) deviations. miRNAs are small noncoding RNAs that work by regulating gene expression at the posttranscriptional level. The entire miRNA group has been shown to regulate over 60% of genes encoding proteins in a human cell. As a part of the RISC complex, miRNA causes inhibition of messenger RNA (mRNA) translation or regulates its degradation [50]. Studies show that dysregulation of miRNAs is well tolerated in normal tissues, but it can influence the function of the tissues experiencing pathological conditions. Changes in the concentration of miRNA molecules have been shown in patients with PAH. The study revealed that decreased expression of miR-124 causes an increased level of polypyrimidine tract-binding protein 1 (PTBP1, also known as hnRNPI), which results in elevated pyruvate kinase (PKM) isoform ratio. The increased PKM2/PKM1 ratio enables anaerobic catabolism, metabolic reprogramming, and enhanced proliferative capacity [50].

Interestingly, it has been proved that pharmacological manipulation of PKM2 activity with miR-124 overexpression or PTBP1 knockdown abolishes the imbalance between the proliferating and eliminated fibroblasts in the pulmonary vascular bed [51].

Despite the numerous studies taking under consideration the miRNA role, a small part of the complex network of gene expression-related relationships has been known to date. Unfortunately, most of these studies did not bring conclusive results that could be used in a new diagnostic and therapeutic approach in patients with PH.

### 3.3. Protein Acetylation and Deacetylation

Subsequent posttranscriptional modification includes histone acetylation and deacetylation. The process is catalyzed by HAT (histone acetyltransferases) and HDAC (histone deacetylases) which modify lysine residues within the N-terminal tail. In consequence of acetylation, the chromatin forms more relaxed structure and promotes gene transcription.

Sirtuin 1 (SIRT1) is one of the HDAC, which via histone deacetylation represses gene expression. The SIRT1 failure was reported in PAH patients' PASMCs, which results in an acetylation/deacetylation imbalance. It augments the PASMC proliferation and leads to vascular remodelling and increased vascular resistance in the pulmonary bed. It was proven, in knockout mice, that deficiency in SIRT1 gene aggravates chronic hypoxia-induced pulmonary hypertension [52].

To reverse this process, the SIRT1 activator resveratrol was used in PAH rats exposed to hypoxia. Interestingly, as a result, the retarded PASMC proliferation and increased apoptosis were noted [53].

Sirtuin 1 also alters mitochondrial metabolism by activating peroxisome proliferator-activated receptor gamma coactivator 1-alpha (PGC-1a), which is a master regulator of mitochondrial biogenesis. It is also an important gluconeogenetic and glycolytic enzyme. SIRT1 deacetylates HIF-1, which represses HIF-1-dependent glycolytic enzymes (i.e., LDH, PFK-1, PGK-1) and glucose transporting proteins (GLUT1, GLUT3). Under anaerobic conditions, the NAD+ concentration (SIRT1 cofactor) decreases, which is followed by the SIRT1 reduction and HIF-1*α* activation [54].

### 3.4. Changes in Phosphoproteome-Kinase Activity

#### 3.4.1. The Src Family of Kinases

The Src Family of Kinases (SrcFKs) constitutes a group of redox-sensitive tyrosine kinases expressed in vascular smooth muscle cells (VSMCs). They contain reactive cysteine residues at physiological pH, and a shift in cellular redox state results in cysteine oxidation, activating SrcFK to stimulate tyrosine phosphorylation. ROS-induced SrcFK activation is postulated to regulate VSMC proliferation and apoptosis [55]. In response to chronic hypoxia, SrcFKs stabilize HIF-1*α* and HIF-2*α* and stimulate prosurvival transcription factors [56]. SrcFKs and their downstream effectors like STAT3 might represent novel therapeutic targets for PAH management.

#### 3.4.2. The Rho-Associated Protein Kinase (ROCK) Pathway (Figure 1)

Rho-associated protein kinase (ROCK) belongs to the AGC (PKA/PKG/PKC) family of serine-threonine kinases and acts as the effector of the G protein Ras homolog family member A (RhoA). It is well established that ROCK participates in regulating vasoconstriction, cellular proliferation, apoptosis, and migration by exerting multidirectional influence on many signalling pathways [57]. It has been shown that Rho kinase concentration and activity are increased in blood cells and pulmonary arteries of patients with PAH compared to controls [58]. It is in line with previous findings from PH-induced animal models. Furthermore, the study by Do et al. [58] revealed a correlation between ROCK activation and PH severity (measured by mPAP) or disease duration. These relations were demonstrated for IPAH, CTD-PAH, and CHD-PAH, but not for CTEPH. Nevertheless, a similar correlation was not observed with respect to cardiac index, right atrial pressure, or plasma BNP concentration [58–61]. Many studies show that the RhoA/ROCK signalling pathway activation leads to vasoconstriction via the Ca^2+^-dependent and Ca^2+^-independent mechanisms. RhoA stimulates ROCK to phosphorylate myosin light chain kinase (MLCK) leading to its subsequent activation. As a result, it leads to accumulation of phosphorylated myosin light chain (P-MLC) and subsequent interaction with actin resulting in smooth muscle cell contraction in a Ca^2+^-independent manner. This process may also affect left ventricular (LV) relaxation and LV filling pressure [62].

Indirectly, the RhoA signalling pathway may also potentiate pulmonary bed vasoconstriction by activating HIF-1*α* in hypoxic conditions. It induces the voltage-gated K+ (Kv) channel expression in PASMCs and leads to depolarization and activation of voltage-operated Ca^2+^ channels (VOCCs). Intracellular Ca^2+^ binds with calmodulin (CaM) and stimulus myosin light chain kinase (MLCK) and induces smooth muscle cell contraction in Ca^2+^-dependent mechanism [61, 63–67].

Wei et al. [68] pointed out that the Rho signalling pathway enhances PAH by stimulating PASMC proliferation via disrupting BMP2/SMAD1 signal transduction. The intact signalling pathway contains BMP2-mediated phosphorylation of SMAD1 at the C-terminal (Ser463/465) and its subsequent translocation into the nucleus. However, it was proved that this process may be interrupt by SMAD1 phosphorylation at the linker region (Ser206) by mitogen-activated protein kinase (MAPK, MEK/ERK) which is one of the downstream signalling molecules of the RhoA/Rho kinase pathway [69]. This alternative SMAD1 modification leads to its ubiquitination and degradation and enhances PASMC proliferation. In the same study [70], ROCK and ERK1/2 inhibitors restored BMP2/SMAD1 function and reduced proliferation level.

It is well known that RhoA is activated by Platelet-Derived Growth Factor BB (PDGF-BB). Tang et al. [69] proved that cells stimulated by PDGF-BB via ROCK and subsequent JNK/c-Jun pathway increase cell cycle protein translation (cyclin D1, CDK2, and CDK4) and promote G0/G1 to S phase passage. It is the other way how the RhoA/ROCK signalling pathway induces PASMC proliferation.

The other dysfunction caused by RhoA that contributes to PH endothelial dysfunction and potentiates disease progression is the decrease in nitric oxide bioavailability. ROCK inhibits eNOS expression via eNOS mRNA destabilization [71]. Additionally, ROCK decreases activity of the PI3K/AKT pathway directly or by PTEN phosphorylation, which in turn leads to reduced eNOS activity and nitric oxide production. It was pointed out that blockage of the RhoA/ROCK signalling pathway is able to restore NO pool [72]. It was proved by using mutant endothelial cells deficient in RhoA. Furthermore, using statins that block HMG-CoA reductase and inhibit geranylgeranyl pyrophosphate production which is necessary for RhoA synthesis presented a similar effect [73]. According to Absi et al., simvastatin may promote vasodilation also by inhibition of Ca^2+^ influx through the L-type of Ca^2+^ channels [74].

## 4. Metabolism Alterations in Pulmonary Hypertension

The up-to-date known mutations and epigenetic gene modifications found in patients with pulmonary hypertension do not provide a complete answer regarding the causes of the disease. This points at the role of regulating pulmonary vascular function affecting further levels of signalling cascades.

### 4.1. Energetic Metabolism in PH

**Figure 2:**
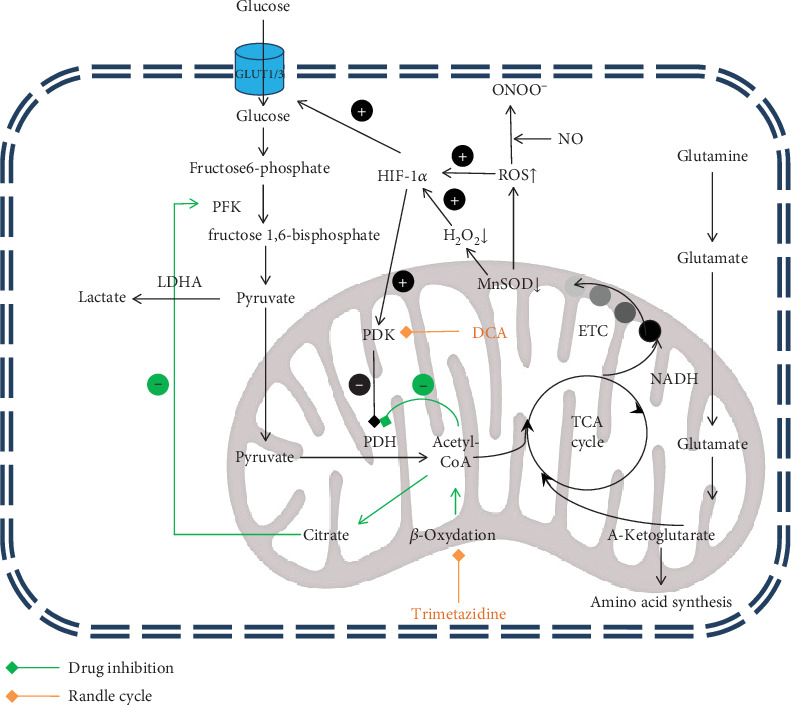
Metabolism alteration in pulmonary hypertension. DCA: dichloroacetate; ETC: electron transport chain; HIF-1*α*: hypoxia-inducible factor; LDHA: lactate dehydrogenase A; MnSOD: mitochondrial superoxide dismutase; PDH: pyruvate dehydrogenase; PFK-6: phosphofructo-1-kinase; ROS: reactive oxygen species; TCA cycle: tricarboxylic acid cycle. Orange arrow: drug inhibition; green arrow: Randle cycle.

Searching for other factors modifying the course of PAH, the deviations in the PAEC metabolism aroused scientists' interest. It was shown that these cells reprogrammed their metabolism by replacing energy acquisition in the Krebs cycle with glycolysis. This phenomenon, known as the Warburg effect, was mostly known as characteristic for autonomic cancer cells. These mutated cells, even in the presence of sufficient oxygen supply, prefer anaerobic respiration to support high proliferation. What is more, the cells are less likely to be exposed to reactive oxygen species. This leads to the “metabolic theory” of PH which indicates dysfunctions in cellular and mitochondrial metabolism as the cause of the disease (Figure 2).

The main product of the glycolysis is a pyruvate. Subsequently, this acid in aerobic conditions is metabolized by PDH (pyruvate dehydrogenase) into acetyl-CoA and enters the tricarboxylic acid cycle (TCA cycle). It was proved that PDH activity is reduced in PAH PAECs and the pyruvate is mostly reduced by lactate dehydrogenase A (LDHA) into lactate. It is in line with the results of few studies, where the increased level of lactate was noted in human plasma and rats' lungs and hearts [75–77]. Additionally, increased transcription and translation of LDHA was proved in the lungs and hearts of MCT-induced PAH rats [75]. The increased level of lactate leads to acidosis which further impairs right ventricle function.

The second metabolic deviation confirmed in PAH cells is increased fatty acid metabolism. Chen et al. pointed out higher levels of fatty acids, L-carnitine, acetyl-L-carnitine, and several long-chain acylcarnitines compared to controls. It may explain partially the decreased activity of PDH. Along with the Randle effect, fatty acid oxidation inhibits glycolysis and glucose transport via pyruvate dehydrogenase and phosphofructokinase suppression. Interestingly, knockout mice deficient in malonyl-coenzyme A gene with reduced fatty acid oxidation do not present pulmonary vasoconstriction and pulmonary arterial hypertension in hypoxic environment [75, 78].

The third metabolic change concerns the glutaminolysis. Normally, this process does not occur in the myocardium or is residual, but it was proved that it is aggravated in hypertrophic hearts. During glutaminolysis, the glutamine is transformed into *α*-ketoglutarate which enters the Krebs cycle. This pathway provides both energy and substrates for the synthesis of nucleic acids, proteins, and lipids, thus compounds necessary for proliferation. The level of glutamine concentration was pointed out as a biomarker of right ventricle hypertrophy which was confirmed in a mouse model [77, 79, 80].

The other important alterations relate to abnormal mitochondrial function. Commonly, mitochondria are considered to be responsible just for generating cells' supply of adenosine triphosphate (ATP); however, their functions are considerably more complex. In PASMCs, they are also oxygen sensors responding to decreased oxygen supply with hypoxic pulmonary vasoconstriction (HPV). It redirects blood to better oxygenated lung areas, while maintaining the appropriate ventilation : perfusion ratio. However, hypoxia reduces mitochondrial reactive oxygen species (ROS) and hydrogen peroxide production. It changes the cell's redox signalling pathways and disrupts metabolic balance. Subsequently, it begins pathological activation of transcription factors like cMyc, forkhead transcription factor, and hypoxia-inducible factor (HIF-1*α*). Finally, it ends with pyruvate dehydrogenase inhibition and metabolic shift in accordance with the Warburg effect [79].

Additionally, some recent studies revealed that PAH patients' mitochondria in pulmonary artery smooth muscle cells are twice more fragmented, when compared to controls. This phenomenon was observed in hypoxia- or monocrotaline-induced PAH rat cells as well. To explain, it should be noted that mitochondria are continuously dividing and joining together which is called fusion and fission and known as mitochondrial dynamics. The more excessive mitochondrial fragmentation is chiefly upregulated by dynamin-related protein 1 (DRP1) which is activated by the cyclin B1/CDK1 which reflects HIF-1*α* stimulation. Extended HIF-1*α* activation and associated mitochondrial fission promote glycolytic shift, proproliferative phenotype, and right ventricle myocyte energy ATP synthesis deviation. What is more, mammalian mitochondria play a key role in initiating apoptosis by releasing various cytotoxic proteins which starts different signal pathways leading to cell death. All the abnormalities that take place in mitochondrial dynamic in PAH PASMC impair apoptosis and aggravate vascular anomalies [81–83].

### 4.2. Regulation of Oxidative Stress: The HIF-1*α* Upregulation in Pulmonary Hypertension

The HIF protein is a heterodimer of the HIF-1*α* or HIF-2*α* and HIF-1*β* subunits. It is a main transcriptional regulator in a process of cells' adaptation activated in response to hypoxia. Under aerobic conditions, HIF-1*α* is not activated because of hydroxylation promoted by hydrolases requiring the presence of oxygen. The HIF-1 is then ubiquitinated which is followed by the proteasome proteolysis. However, in hypoxia, the HIF-prolyl hydroxylases (HPHs) are blocked, which leads to stabilization of the HIF-1*α* subunit with a subsequent increase in its cellular concentration. This in turn results in transcriptional activation of over 40 metabolism-regulating genes [84].

HIF-induced transcriptional activity is aimed at restoring the optimal oxygen supply by modifying genes, whose products regulate vascular tone and stimulate angiogenesis and erythropoiesis.

Several studies have shown the twofold higher HIF-1*α* expression in epithelial cells of patients with IPAH when compared to the control group. The levels of this protein were higher in the cells of patients with IPAH even under normoxia. Its higher expression in plexiform lesions was also shown, which indicates the role of HIF-1*α* in promoting the proliferative vasculopathy of IPAH [85].

Higher concentration of plasma HIF-1*α* may be explained by “pseudohypoxic” environment found in PAH mitochondria. The reason for that is the mitochondrial superoxide dismutase (MnSOD) dysfunction found in PH. MnSOD is normally responsible for scavenging reactive oxygen species (ROS) and converting them into hydrogen peroxide removed further by catalase or glutathione peroxidase (GPx). The decreased level and activity of MnSOD2 pointed out in PAH (mostly according to epigenetic silencing) resulted in reduced hydrogen peroxide (H_2_O_2_) production, which creates hypoxia-like status that activates HIF-1*α* [86]. Furthermore, reactive oxygen species have been shown to stabilize the HIF-1*α* and increase its concentration.

This hypothesis is in line with studies in which the overexpression of human extracellular superoxide dismutase (EC-SOD) in rats with monocrotaline- (MCT-) induced pulmonary hypertension significantly reduces pulmonary hypertension development and SMC proliferation [87, 88].

Reactive oxygen species also affect nitric oxide (NO) metabolism. It is synthesized by ECs and is one of the key factors regulating pulmonary vascular reactivity, which affects also SMC proliferation and migration. Furthermore, it is well known that the bioavailability of nitric oxide is lower in patients with PH, and the disturbance in that signalling pathway is one of the components leading to the development of PH [89].

Increased oxidative stress and ROS concentration contribute to the severity of pulmonary hypertension by potentiating superoxide anion with nitric oxide reaction. It results in peroxynitrite anion formation. As a result, the pool of available nitric oxide is reduced. This is consistent with observations that the pulmonary pool as well as the total body NO is lower in IPAH patients, as compared with healthy controls. Indeed, studies showed that in EC cells in which the SODs were knocked down by RNA interference, the NO production was decreased [31].

HIF-1*α* induces also metabolic changes mentioned above by upregulating PDK expression, which results in pyruvate dehydrogenase kinase inhibition leading to glycolytic shift. HIF-1*α* also enhances the Warburg effect by activating glycolysis genes, GLUT1 and GLUT3 transporters, and by regulating the cellular pH [86]. Moreover, HIF-1*α* is also capable of inhibiting the hypoxia-induced cell death by limiting hypoxia-induced p53 phosphorylation. It also increases the rate of cell proliferation by induction of differentiation inhibitors. This results in the expansion of less differentiated cells with a higher mitotic potential. Their spread is further facilitated by increased expression of extracellular enzymes [84, 90].

## 5. Novel Biomarkers in Pulmonary Hypertension Diagnosis: Results from the Genomic, Transcriptomic, Proteomic, and Metabolomic Studies

As long as we are not able to treat pulmonary hypertension successfully, studies were also focused on identifying the new risk factors and risk assessment methods, so as to more accurately specify the group of patients requiring intensive supervision.

One of the most common risk assessment methods used to predict survival in patients with pulmonary arterial hypertension is the Registry Risk Score for Pulmonary Arterial Hypertension scale (REVEAL scale). It provides a risk assessment tool but requires using a variety of testing procedures including invasive diagnostic methods [91]. For this reason, the new noninvasive biomarkers assessing the severity and predicting a more advanced course of the disease which will be able to distinguish patients requiring more intensive management strategies are also sought.

### 5.1. Biomarkers Already Used in Clinical Practice

One of the paramount plasma biomarkers well established in PH are brain natriuretic peptide (BNP) and N-terminal brain natriuretic prohormone BNP (NT-proBNP). Simpson et al. were focused on finding the correlation among NT-proBNP, the plasma concentration of soluble suppression of tumorigenicity 2 (sST2) protein, and the disease severity or survival rate in group 1—the PAH patients. Both markers revealed to be useful as predictors for future mortality and disease severity. The primary sensitivity of NT-proBNP assessed in follow-up was greater than that of BNP. Contrary to Simpson et al., Cavagna and colleagues consider BNP as more specific (90% vs. 87%) and sensitive (60% vs. 45%) [92, 93]. BNP appears to be more specific in distinguishing PH patients, as far as it is less susceptible to elevation caused by comorbidity of renal dysfunction frequent in this population. On the other hand, NT-proBNP seems to be superior to BNP as a mortality predictor [94].

The usefulness of implementing the NT-proBNP to evaluate the risk of PH was also confirmed in the DETECT study. The plasma level of NT-proBNP is one of the components in the DETECT algorithm, which identify the subgroup of patients with systemic sclerosis at risk of PH that should be referred for echocardiography and subsequently in some cases for right heart catheterization (RHC) [95]. Furthermore, incorporating the sST2 and NT-proBNP data into the REVEAL scale improved its effectiveness [92]. In line with Simpson et al., Mirna et al. confirmed sST2 to be an effective general PH biomarker irrespective of PH subgroup [96]. At this point, it is worth noting that sST2 may be also elevated in other disease such as heart failure, myocardial infarction, or acute aorta dissection [97–100].

The second marker incorporated to the DETECT algorithm is uric acid (UA) level. It was confirmed that patients with PH have elevated level of UA which was connected with increased odds of PAH diagnosis at RHC in these patients. A UA level > 6.2 mg/dL was linked with fourfold higher chance of PAH diagnosis. One UA serum unit (also within normal limits) was found to raise mortality risk in PAH by 14%. It was also associated with disease severity. As with BNP level, combining UA with other noninvasive biomarkers increased diagnostic possibilities working synergistically. Furthermore, after incorporating vasodilator therapy, the UA level drops, which was associated with a reduction in total pulmonary resistance and improved survival [101–103].

The answer regarding pathophysiology of UA level and PAH patients' outcomes was partially characterized in the hypoxia-induced PH rat model, where increased xanthine oxidoreductase (XOR) activity was linked with hypoxic exposure, right ventricle hypertrophy, and pulmonary vascular remodelling. Additionally, UA decreases the NO and cGMP production in PAECs by activating arginase and promoting its attachment with L-arginine [101, 104].

Some other studies show a similar prognostic usefulness present in cardiac troponin (cTnT) or high-sensitivity troponin (hsTnT) wherein the elevated level was in line with worse prognosis and increased risk of hemodynamic destabilization. The troponin value over 30 pg/mL was strongly correlated with death within 12 months. Heresi et al. indicated higher hsTnT than cTnT sensitivity and proved that hsTnT is more precise in WHO functional class ≥ 2 patient identification than NT-proBNP or H-FABP. An elevated hsTnT level also corresponded with systolic RVC dysfunction, worse echocardiographic parameters (larger right atrial area), and worse 6 min walk distance (6-MWD) results [105–107].

### 5.2. Biomarkers Being in the Study Phase and Capable of Showing Some Clinical Utility

Simultaneously, Mirna et al. indicated heart-type fatty acid binding protein (H-FABP), soluble urokinase-type plasminogen activator receptor (suPAR), and growth differentiation factor-15 (GDF-15) as more specific molecules. Different levels of all pointed-out biomarkers are characteristic for postcapillary PH (group 2), while H-FABP may be also increased in pulmonary hypertension that is associated with lung disease and/or hypoxia (group 3). This would make these indicators useful for determining the causes of the disease [96].

The group of new biomarkers that may be used to distinguish a patient with COPD with comorbidity of secondary PH includes inflammatory indexes such as platelet-to-lymphocyte ratio (PLR), neutrophil-to-lymphocyte ratio (NLR), and systemic immune-inflammation index (SII). Zuo et al. proved that all these indexes are significantly higher in a patient with acute exacerbation and PH compared to the control group just with acute exacerbation of COPD. NLR was characterized by higher discriminative ability than PLR and SII. It is also mentioned to be a systemic inflammation indicator associated with the worse prognosis [108].

Osteopontin (OPN), a small glycoprotein, may also be useful to evaluate PH progression. Its elevated level is considered to be connected with myocardial dysfunction and also acts as modulating factor of proliferation and remodelling in pulmonary bed cells. It is an additional risk factor of mortality in PAH. Simultaneous elevated levels of NT-proBNP and OPN in patients with PAH were associated with an 11-fold higher risk of death compared to patients with normal biomarker results [109, 110].

According to the metabolic shift described above, the composition of components essential to sustain altered metabolic pathways is different in PH patients. The elevated level of TCA and glycolysis intermediates as well as nucleosides and ketone bodies was found. After comparing diabetes mellitus, heart failure, and PAH patients' metabolome, Rafikov et al. distinguished eleven metabolites that may be useful in patient screening which are considered to be PAH metabolic fingerprint. As they noticed in their studies, the unique metabolic biomarkers that they characterized are specific for PAH patients and additional metabolic profiling should be performed for patients from other WHO PH groups [111].

Evaluating specific circular RNA (circRNA) was also described as an effective mechanism for predicting the IPAH. Circular RNA is a group of noncoding RNAs that was primarily considered to be a “junk” genetic material. A characteristic feature is back-splicing while two sites of RNA are linked together forming the final molecule [112]. Zhang et al. found that the circRNA (circ_0068481) level is significantly higher in patients with IPAH. As far as it has circular structures, it is more stable and resistant from nuclease degradation which makes it a better serum biomarker. The circ_0068481 was shown to have high sensitivity and specificity (respectively, 74.39% and 98.78%) in distinguishing PAH patients. A more elevated level was found in case of comorbidity of IPAH and right ventricular failure and in a patient who eventually died in a short time because of IPAH. It suggests circ_0068481 as a biomarker of poor outcome [113]. Additionally, the role of circRNA as a biomarker of CTEPH was also postulated. It was revealed that some circRNA may have an important role in pathogenesis via interaction with specific miRNAs [114].

Arvidsson et al. revealed that extracellular matrix (ECM) whose role in the pathogenesis of PH was confirmed could also play an important role in diagnosing patients. ECM takes part in vascular remodelling, leads to wall stiffness, and favours proliferation in PH. It was proved that the MMP-7 level is significantly higher compared to that in the healthy control group and lower in PAH than that in other WHO PH groups, which indicates in turn that MMP-7 may be a useful tool in allocating patients in each subgroup. The MMP-7 sensitivity and specificity were estimated to be 58.7% and 83.3%, respectively, in distinguishing patients with PAH from all patients with dyspnoea. In the same study, the metalloproteinase-2, metalloproteinase-7, metalloproteinase-9, and metalloproteinase-12 levels were also increased in PAH samples but the meaning of these results needs further investigation [115].

Noteworthily, there are also several other biomarkers including circulating angiogenic modulatory factors (VEGFR1, CRP, endostatin, or PCEB-ACE) or inflammatory markers (galectin-3, GDF-15) which were related to the pathophysiology of pulmonary hypertension. However, due to the relatively low specificity, in our opinion, they cannot find the usefulness in clinical practice.

## 6. Novel Drug Targets Modifying the Energetic Metabolism Alterations and Limiting the Posttranslational Modifications

### 6.1. Targeting Energetic Metabolism

As the metabolic mechanisms underlying PAH were explored, numerous studies were initiated to restore physiological signalling pathways. To recover the PDH function, dichloroacetate (DCA), a PDK inhibitor, was used. DCA is a pyruvate analogue that increases mitochondrial-dependent apoptosis in abnormal pulmonary artery cells not affecting other vessels and normal cells. It increases cytochrome c level and hydrogen peroxide (H_2_O_2_) production. H_2_O_2_ higher concentration is a result of PDH and electron transport chain restoration made by DCA. Cytochrome c and H_2_O_2_ lead to potassium voltage-dependent (Kv) channel activation and Kv1.5 expression upregulation. The increased outflow of potassium ions and cytochrome c interaction with caspase 3 promote DCA-dependent apoptosis. The other way that H_2_O_2_ may promote apoptosis is enhancing the likelihood of mitochondrial permeability transition pore (MTP) opening. MTPs increase mitochondrial membrane permeability and lead to mitochondrial swelling, which results in membrane injury and cytochrome c upconcentration [82, 116, 117].

It was proved in preclinical studies that oral DCA improves myocyte contractility, right ventricular function, and cardiac electrical remodelling. Its activity is limited to abnormal or proliferating PASMC which minimalizes the toxicity. DCA was used in patients with glioblastoma multiforme in a long-term clinical study, and now, the usefulness of DCA treatment is being checked in PAH [78, 82, 116, 117].

The second drug with potentially beneficial effects in PAH therapy is trimetazidine—a selective inhibitor of fatty acid beta-oxidation. It reduces activity of the mitochondrial enzyme long-chain 3-ketoacyl coenzyme A thiolase (3-KAT). Blocking thiolase, in line with the Randle cycle and reciprocal relationship between fatty acid oxidation and glucose oxidation, results in increased glucose oxidation. Additionally, trimetazidine restores intracellular phosphocreatine resources, thereby optimizing cell metabolism, and acts cytoprotectively. The trimetazidine effect on cardiomyocytes is to prevent adenosine triphosphate (ATP) deficiency, to reduce cellular sodium and calcium accumulation, and in this way to decrease the intracellular acidosis. Trimetazidine has also been shown to have beneficial effects on remodelling and improving left ventricular ejection fraction [118, 119].

The other promising target for drugs that may be used in PAH treatment is mitochondrial fission inhibition. By decreasing the DRP1-dependent signalling pathway with mitochondrial division inhibitor 1 (Mdivi-1), the PASMC proliferation rate is reduced and right ventricle myocyte energy ATP synthesis is restored. Mdivi-1 causes cell cycle arrest in the G2/M mitotic phase. The antiproliferative effect of Mdivi-1 was already proved in monocrotaline- and hypoxia-induced pulmonary hypertension in human PAH PASMCs and in rodent models [79, 81, 82].

### 6.2. Targeting Oxidative Stress and Inflammation

Luo et al. characterized the new CD146-HIF-1*α* signalling pathway which might become an interesting therapeutic target. CD146 is a cell adhesion molecule found in the vascular wall that plays a role in PASMC differentiation, migration, and proliferation. It is found in the crosstalk of signalling pathways such as hypoxic, PDGF-BB/PDGFR*β* and the Notch one. All of them are widely recognized as relevant in PAH pathogenesis and are pointed to be promising avenues in PAH treatment. Disruption of the CD146-HIF-1*α* axis leads to limiting PAH progression but does not reverse the dysfunctions completely [120].

Other studies indicate the possibility of using doxycycline, a matrix metalloproteinase and angiogenesis inhibitor. Additionally, interferon 2*α* might be used as a proliferation and collagen synthesis inhibitor and a macrophage and immune system modulator [121].

### 6.3. Therapeutic Implications of MicroRNA in Pulmonary Hypertension

Since numerous studies show that dysregulation of miRNAs in the pulmonary vasculature results in abnormalities in gene expression and contributes to the pathogenesis of PAH, the restoration of miRNA expression to physiological levels may constitute an interesting approach for managing pulmonary hypertension, especially PAH. This may be obtained using the anti-miRNA (anti-miR) oligonucleotide-based and miRNA mimic-based approaches. Nevertheless, the administration of miRNA should be targeted to the specific vascular cells (endothelial cells, vascular smooth muscle cells, and fibroblasts), in order to minimize any off-target effects on other cells. Moreover, the dose of anti-miR or miRNA mimics delivered should be considered carefully in order to avoid the off-target effects, which requires developing the vascular cell-specific delivery methods along with techniques providing regulated miRNA release [122].

Interestingly, a recent study by Chen et al. [123] demonstrates that dysregulation of the dynamin-related protein 1 adapter proteins and mitochondrial dynamic protein of 49 and 51 kDa (MiD49 and MiD51) increases mitotic mitochondrial fission and promotes pulmonary arterial hypertension. What is more, the authors have shown that silencing MiDs causes cell cycle arrest through an ERK1/2- and CDK4-dependent mechanism, decreases cell proliferation rate, and increases apoptosis. In an experimental animal model, nebulizing miR-34a-3p or siMiDs resulted in regression of pulmonary hypertension which could constitute a novel therapeutic strategy, based on a molecularly defined new drug target.

### 6.4. Kinase Activity as a Therapeutic Target in Pulmonary Hypertension

Kinases hold a promise to become potential drug targets in pulmonary hypertension.

The Rho kinase activation in PAH sensitizes to calcium resulting in greater vasoconstriction which is not reversed by conventional vasodilators and likely contributes to vascular stiffening [124]. Rho kinase inhibitors, such as fasudil, have been studied in human PAH cohorts [125], and the results suggest that they might be effective and safe in PAH patients with right ventricular failure [126, 127]. Fasudil was recently also demonstrated to be effective in the short-term treatment of subjects with pulmonary hypertension resulting from left ventricular failure with preserved ejection fraction (HFpEF) [62]. Although the elimination half-life of a drug is short, recent studies are guided to extend its working time. Although fasudil appears to be a promising drug in PH treatment, further trials are needed to evaluate the long-term effects of its use.

Considering the role of SrcFK in promoting ROS-induced vascular remodelling, targeting SrcFK holds some therapeutic promise in PAH [128]. Imatinib, a tyrosine kinase inhibitor which is commonly used in the therapy of chronic myeloid leukaemia (CML) due to its inhibitory action on the chimeric BCR-ABL tyrosine kinase, is also a nonspecific inhibitor of the PDGF receptor and may reverse PH, as demonstrated in an experimental model [129]. Significant improvement in 6-MWD in patients receiving imatinib was found in the randomized double-blind placebo-controlled IMPRES trial. In this study, imatinib improved exercise capacity and hemodynamics in patients with advanced PAH, but serious adverse events and study drug discontinuations were common [130]. Interestingly, dasatinib, another BCR-ABL and PDGF receptor blocker was, demonstrated to increase PAH development when used in patients with CML [131].

### 6.5. Modification of Protein Nitration/S-Nitrosylation as a Therapeutic Approach in PAH Management

Preventing some posttranslational modifications, as it results from the proteomic studies, may also limit the progression of pulmonary hypertension [132]. The nitration of tyrosine to 3-nitrotyrosine is an oxidative modification of tyrosine by nitric oxide and is associated with many diseases, and targeting of protein kinase G- (PKG-) I represents another potential therapeutic strategy for pulmonary hypertension [133]. What is more, some data suggests that inactivation of the RhoA by nitric oxide occurs via S-nitrosylation, which suggests that posttranslational modification and inactivation of RhoA by S-nitrosylation constitute a signalling mechanism that contributes to the regulation of vascular SMC proliferation [134].

## 7. Conclusions

To date, numerous studies on the aetiology and pathophysiology of pulmonary hypertension at molecular level were conducted. Simultaneously, some clinical studies have shown potential usefulness of some well-known and widely recognized cardiovascular biomarkers, such as BNP, NT-proBNP, hsTnT, sST2, osteopontin, or serum uric acid, in diagnosing and prognosing the outcome of pulmonary hypertension. The enthusiasm from their potential clinical usefulness in diagnosis and management of pulmonary hypertension is cooled down by their low specificity accompanied with numerous cardiovascular comorbidities of PH subjects. On the other hand, a large body of basic research studies provides us with novel molecular pathomechanisms, biomarkers, and drug targets, according to the evidence-based medicine principles. Unfortunately, the simple implementation of these results to clinical practice is impossible due to a large heterogeneity of the pathophysiology of PH, where the clinical symptoms constitute only a common denominator and a final result of numerous crosstalking metabolic pathways.

Therefore, future studies, based mostly on translational medicine, are needed in order to both organize better the pathophysiological classification of various forms of pulmonary hypertension and define precisely the optimal diagnostic markers and therapeutic targets in particular cases of PH.

## Figures and Tables

**Figure 1:**
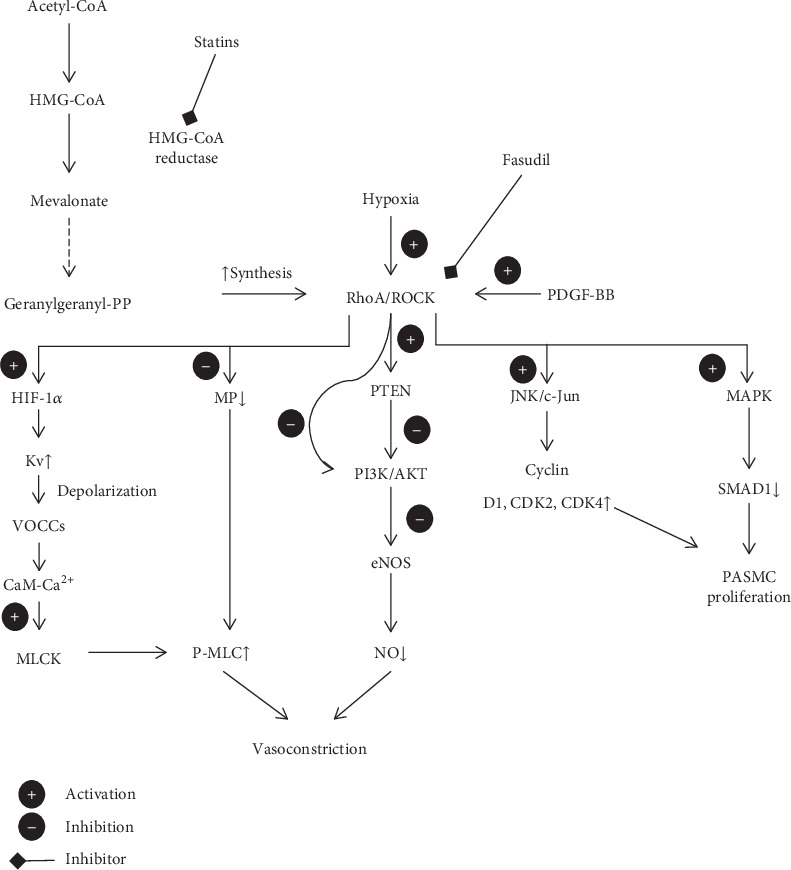
RhoA/ROCK signalling pathway in PH pathogenesis. CaM: calmodulin; eNOS: endothelial nitric oxide synthase; Kv: voltage-gated K+ channels; MAPK: mitogen-activated protein kinase; MLCK: myosin light chain kinase; MP: myosin phosphatase; NO: nitric oxide; PASMC: pulmonary arterial smooth muscle cell; PDGF-BB: Platelet-Derived Growth Factor BB; P-MLC: phosphorylated myosin light chain; PTEN: phosphatase and tensin homolog; RhoA: G protein Ras homolog family member A; ROCK: Rho-associated protein kinase; VOCCs: voltage-operated Ca^2+^ channels. (+) activation, (−) inhibition, and (←) inhibitor.

**Table 1:** Comprehensive clinical classification of pulmonary hypertension (updated from Simonneau et al. [3]).

1. Pulmonary arterial hypertension (PAH)	1.1. Idiopathic	
	1.2. Heritable	1.2.1. BMPR2
		1.2.2. ALK1, ENG, SMAD9, CAV1, KCNK3
		1.2.3. Unknown
	1.3. Drug and toxin induced	
	1.4. Associated with the following:	1.4.1. Connective tissue diseases
		1.4.2. Human immunodeficiency virus (HIV) infection
		1.4.3. Portal hypertension
		1.4.4. Congenital heart diseases
		1.4.5. Schistosomiasis
1′. Pulmonary veno-occlusive disease (PVOD) and/or pulmonary capillary hemangiomatosis (PCH)	1′.1. Idiopathic	
	1′.2. Heritable	1′.2.1. EIF2AK4 mutation
		1′.2.2. Other mutations
	1′.3. Drug, toxin, and radiation induced	
	1′.4. Connective tissue diseases	
	1′.5. Human immunodeficiency virus (HIV) infection	
1^″^. Persistent pulmonary hypertension of the newborn (PPHN)		
2. Pulmonary hypertension due to left heart disease	2.1. Left ventricular systolic dysfunction	
	2.2. Left ventricular diastolic dysfunction	
	2.3. Valvular disease	
	2.4. Congenital/acquired left heart inflow/outflow tract obstruction and congenital cardiomyopathies	
3. Pulmonary hypertension due to lung disease and/or hypoxia	3.1. Chronic obstructive pulmonary disease	
	3.2. Interstitial lung disease	
	3.3. Other pulmonary diseases with mixed restrictive and obstructive pattern	
	3.4. Sleep-disordered breathing	
	3.5. Alveolar hypoventilation disorders	
	3.6. Chronic exposure to high altitude	
	3.7. Developmental abnormalities	
4. Chronic thromboembolic pulmonary hypertension (CTEPH)		
5. Pulmonary hypertension with unclear multifactorial mechanisms	5.1. Hematologic disorders: chronic haemolytic anaemia, myeloproliferative disorders, splenectomy	
	5.2. Systemic disorders: sarcoidosis, pulmonary histiocytosis, lymphangioleiomyomatosis (LAM)	
	5.3. Metabolic disorders: glycogen storage disease, Gaucher disease, thyroid disorders	
	5.4. Others: tumoral obstruction, fibrosing mediastinitis, chronic renal failure on dialysis, segmental PH	

BMPR2 = bone morphogenetic protein receptor type 2; EIF2AK4 = eukaryotic translation initiation factor 2 alpha kinase 4.
